# Case report: Clinical, genetic and immunological characterization of a novel *XK* variant in a patient with McLeod syndrome

**DOI:** 10.3389/fgene.2024.1421952

**Published:** 2024-08-21

**Authors:** Christine Anna Dambietz, Andrea Doescher, Michael Heming, Anja Schirmacher, Bernhard Schlüter, Andrea Schulte-Mecklenbeck, Christian Thomas, Heinz Wiendl, Gerd Meyer zu Hörste, Sarah Wiethoff

**Affiliations:** ^1^ Department of Neurology with Institute of Translational Neurology, University Hospital Münster, Münster, Germany; ^2^ DRK Blutspendedienst NSTOB, Institute Bremen-Oldenburg, Springe, Germany; ^3^ Central Laboratory, University Hospital Münster, Münster, Germany; ^4^ Institute of Neuropathology, University Hospital Münster and University of Münster, Münster, Germany

**Keywords:** *XK* gene, McLeod syndrome, neuropathy, myopathy, Kell system, neurogenetic disease, intrathecal immunity, flow cytometry

## Abstract

**Introduction:** Pathogenic variants in the *XK* gene are associated with dysfunction or loss of XK protein leading to McLeod syndrome (MLS), a rare X-linked neuroacanthocytosis syndrome with multisystemic manifestation. Here we present clinical, genetic and immunological data on a patient originally admitted to our clinic for presumed post-COVID-19 syndrome, where thorough clinical work-up revealed a novel frameshift deletion in *XK* causal for the underlying phenotype. We additionally review the clinicogenetic spectrum of reported McLeod cases in the literature.

**Methods:** We performed in-depth clinical characterization and flow cytometry of cerebrospinal fluid (CSF) in a patient where multi-gene panel sequencing identified a novel hemizygous frameshift deletion in *XK*. Additionally, Kell (K) and Cellano (k) antigen expression was analysed by Fluorescence-activated Cell Sorting (FACS). *KEL* gene expression was examined by RNA sequencing.

**Results:** A novel hemizygous frameshift deletion in the *XK* gene resulting in premature termination of the amino acid chain was identified in a 46-year old male presenting with decrease in physical performance and persisting fatigue after COVID-19 infection. Examinations showed raised creatine kinase (CK) levels, neuropathy and clinical features of myopathy. FACS revealed the K-k+ blood type and reduced Cellano density. CSF flow cytometry showed elevation of activated T Cells.

**Conclusion:** In-depth clinical, genetic, immunological and ribonucleic acid (RNA) expression data revealed axonal neuropathy, myopathy and raised levels of activated CSF-T-lymphocytes in a patient with a previously unpublished frameshift deletion in the *XK* gene.

## Introduction

**FIGURE 1:**
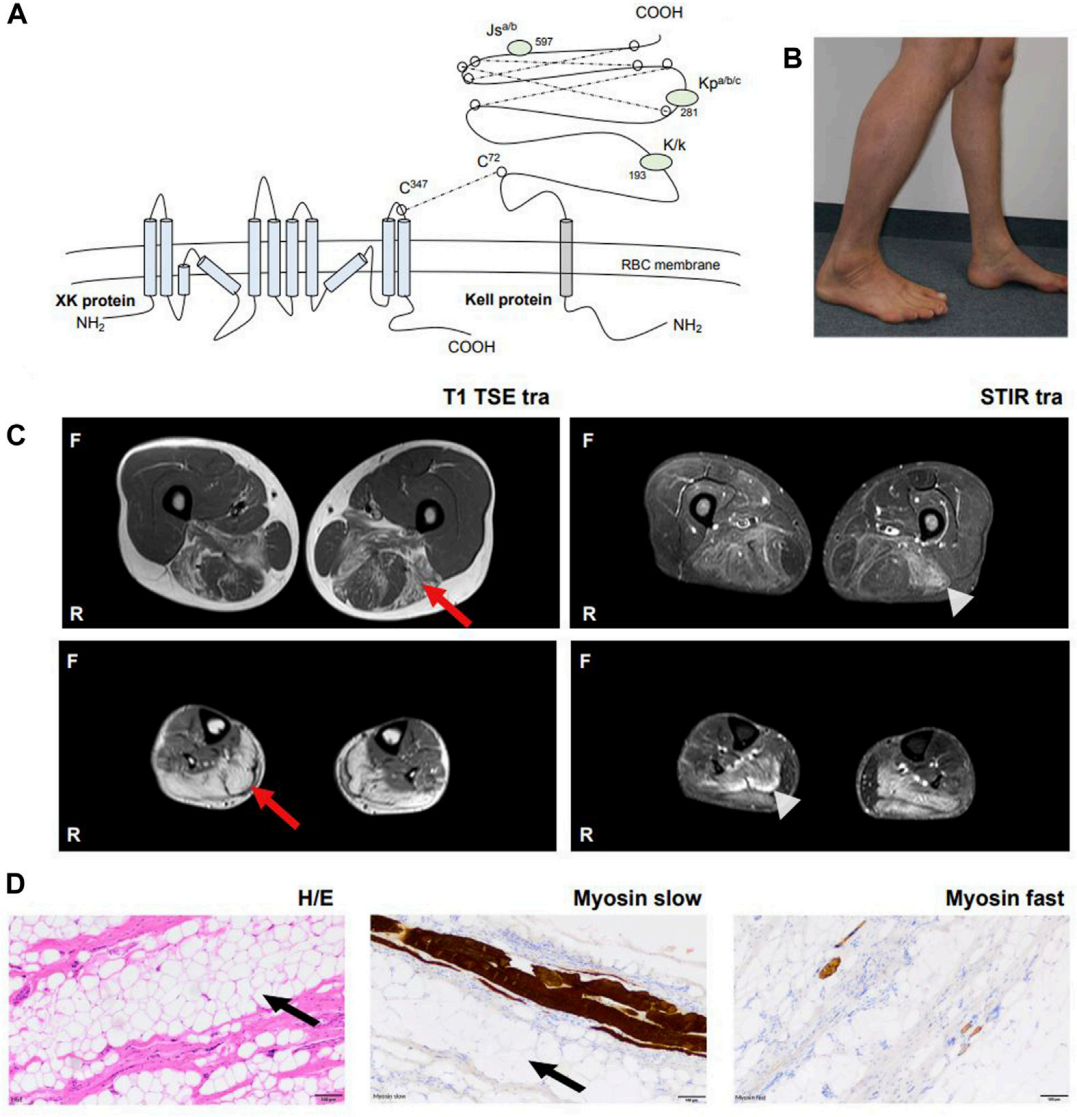
Overview of *XK* pathophysiology and clinical manifestation in our patient. **(A)** The *XK* gene codes for the XK protein which consists of ten transmembrane domains and forms a heterodimer with the Kell protein via a disulfide bond (Cys^347^_XK_ – Cys^72^_Kell_). The XK protein is expressed in many tissues, including RBCs, bone marrow, brain and other neuronal tissues. The *KEL* gene codes for the Kell protein, which is a glycoprotein of 732 amino acids. The Kell protein is expressed on RBCs, bone marrow, testes and fetal liver. The protein expresses 25 antigens, the best known Kell blood group antigens are Kell (K) and Cellano (k) located at amino acid position 193, Kp at amino acid position 281 and Js at amino acid position 597. **(B)** Photograph. The patient’s lower legs with dominant atrophy of the left gastrocnemius muscle. **(C)** Whole body muscle MRI. Bilateral fatty oedematous atrophy of adductors, hamstring muscles and calves with T1 hyperintensity (red arrow). Hyperintensity in STIR sequences revealed recent oedema in the above muscles (light grey arrowhead). Muscles in other parts of the body were not affected. **(D)** Histological examination after biopsy of the left gastrocnemius muscle with hematoxylin and eosin staining, myosin slow and myosin fast staining revealed severe atrophy of skeletal muscle with vacant fat (black arrow) and surplus of connective tissue, but without signs of inflammation, rimmed vacuoles or ragged red fibers. No other specific structural or immunohistochemical abnormalities were detected. Abbreviations: Cys, cysteine; MRI, Magnetic resonance imaging; F, front; H/E, hematoxylin eosin staining; R, righthand side; RBCs, red blood cells; STIR, Short Tau Inversion Recovery sequence.

Pathogenic variants in the *XK* gene are associated with MLS, a rare X-linked neuroacanthocytosis syndrome affecting mainly males (Roulis et al., 2018). Mainly private pathogenic variants (small deletions, frameshift variants, missense variants and insertions) have been identified in *XK* (Jung et al., 2004) as well as extended deletions spanning beyond the *XK* gene (Roulis et al., 2018). The *XK* gene, located in the Xp21.1 region of the X-chromosome, codes for the transmembrane XK protein which forms a heterodimer with the Kell protein (Figure 1A). It is known to express the antigen Kx on red blood cells (RBCs) and support the expression of Kell protein antigens. Recent evidence suggests XK forming a complex with chorein/*VPS13A*, a likely hint for shared pathophysiological mechanisms in clinical resemblance of MLS and *VPS13A* disease (chorea acanthocytosis) (Peikert et al., 2022). Identified genetic changes in *XK* result in dysfunction or loss of the XK protein which is associated with changes in skeletal structure of different cells, e.g., erythrocytes (Ballas et al., 1990; Lee et al., 1991; Terada et al., 1999). Clinical findings associated with *XK* variants comprise a multisystemic spectrum disorder including acanthocytosis, haemolysis, anaemia, spleno- and hepatomegaly, cardiomyopathy, myopathy, neuropathy, dystonia, choreatic movement disorders as well as neuropsychiatric disorders and cognitive decline. Symptoms usually manifest in males around their fourth decade and progress slowly. Females can rarely be affected, generally displaying a milder phenotype (Danek et al., 2001; Roulis et al., 2018).

In this case report we present the clinical, genetic and immunological characterization of a patient with a yet unpublished, but pathogenic deletion in *XK*. We display the restriction of Kell antigen expression resulting from the identified deletion to support its pathogenicity, and we review 44 further cases identified from the literature with multisystemic MLS adding to a better understanding of this rare disease with variable phenotypic presentation.

## Methods

### Molecular genetic analysis

Genetic analysis of blood was performed using a customised myopathy multi-gene panel [Twist Version one analysed with varvis^®^ (version 1.19)] covering candidate genes for myopathy and muscular dystrophy. Evaluation of results, clinicogenetic correlation and classification of variants followed the four eyes principle and *American College of Medical Genetics and Genomics* (ACMG) guidelines.

### Fluorescence-activated Cell Sorting (FACS) and flow cytometry

To analyse expression of Kell protein antigens Kell (K, K1) and Cellano (k, K2) on RBCs, FACS analysis of whole blood was performed with anti-K and anti-k antibodies. Additional flow cytometry of cerebrospinal fluid (CSF) was performed to analyse the subcomposition of cells in the CSF.

### Rh flow cytometry

Additional FACS was performed to analyse the expression of Kell protein antigens Kell (K) and Cellano (k) on RBCs.

### Gene expression analysis

Analysis of *KEL* gene expression was performed by real-time polymerase chain reaction (real-time PCR) using a validated expression assay (TaqMan™ Gene Expression Assay) with specific primers and StepOne™^T^ software (version 2.2.2. Applied Biosystems 2011) for RNA quantification analysis. Detailed methods including primer sequences and a list of genes included in the myopathy multi gene panel can be found in Supplementary Data S1.

### Case description

**FIGURE 2:**
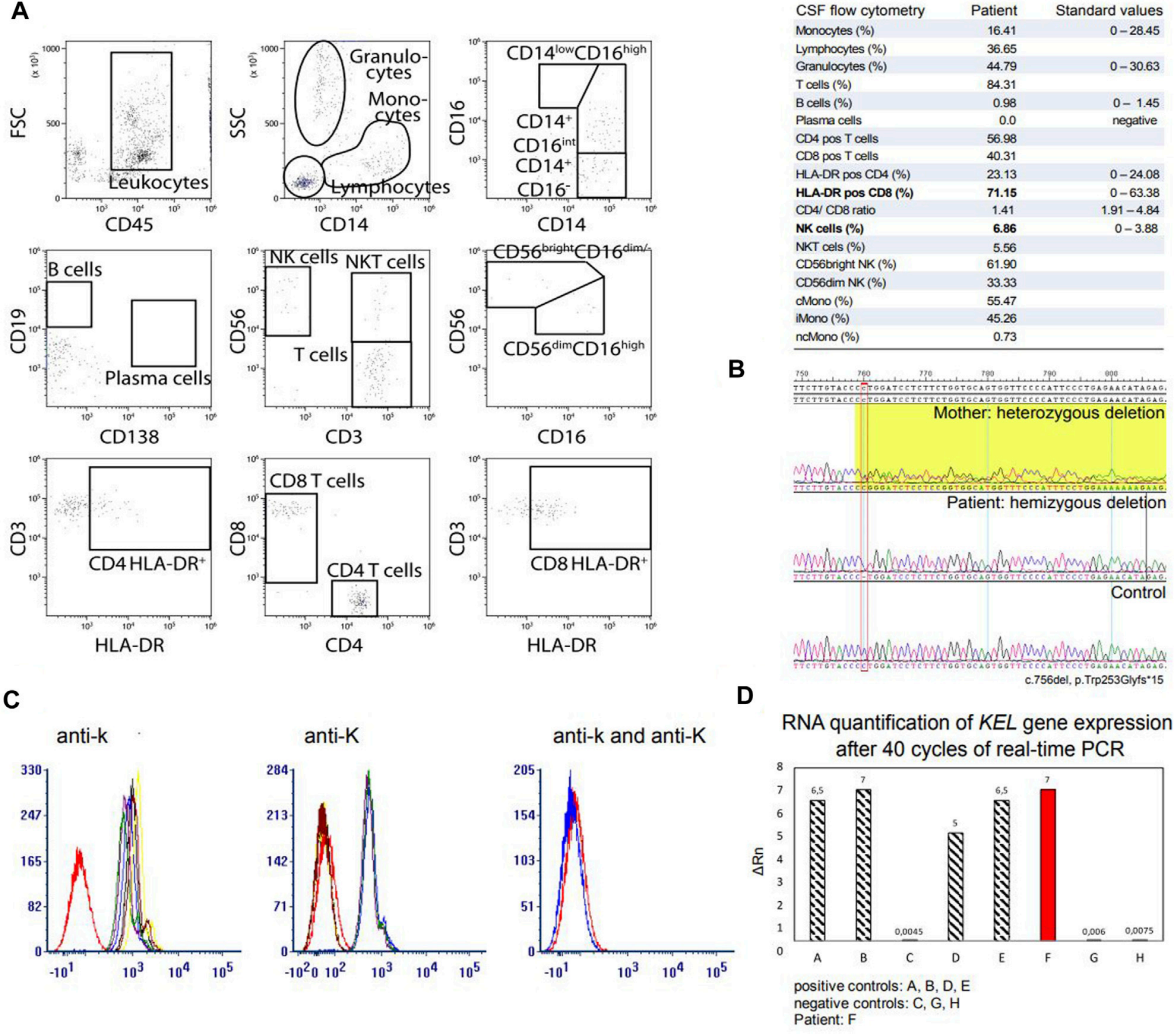
CSF flow cytometry analysis, molecular genetic analysis and *KEL* gene expression analysis in our patient. **(A)** CSF flow cytometry. Flow cytometry analysis of the patient’s CSF revealed elevation of HLA-DR+CD8+ T cells with a CD4/CD8 ratio of 1.4 and elevation of NKs. **(B)** Sanger sequencing of the *XK* gene revealed a novel deletion at position 756, resulting in a frameshift deletion and a premature termination of the amino acid chain (exon 3; c.756del, p.Trp253Glyfs*15). Segregation analysis in the mother revealed that they carry the same *XK* deletion. **(C)** Fluorescence-activated Cell Sorting was performed to analyse the expression of Kell antigens Kell (K) and Cellano (k) on erythrocytes. Five samples of fresh EDTA and two samples of RBCs were detected with antibodies (a) anti-k and (b) anti-K. Our patient is marked in red. (c) Presentation of the Kell antigens Cellano (k) and Kell (K) in our patient. **(D)** *KEL* gene expression assay. Real-time PCR was performed using the TaqMan Gene Expression Assay. Our patient with the novel *XK* deletion (F) showed no restriction in expression of the *KEL* gene on RNA level. Abbreviations: CD56^bright^ NK, CD56^bright^ natural killer cells; CD56^dim^ NK, CD56^dim^ natural killer cells; cMono, classical monocytes; CSF, cerebrospinal fluid; del, deletion; EDTA, Ethylenediaminetetraacetic acid; Gly, glycine; HLA-DR pos, activated cells; iMono, intermediate monocytes; ncMono, non-classical monocytes; NK, natural killer cells; NKT, natural killer T cells; PCR, polymerase chain reaction; RBCs, red blood cells; RNA, ribonucleic acid; Trp, tryptophane.

A 46-year old male patient presented to the neurology department of the University Hospital Münster with muscular weakness, decreasing physical performance and exercise intolerance following mild coronavirus disease 19 (COVID-19). Prior to presentation, physical examination, laboratory tests, electrocardiogram, echocardiography, body plethysmography, lung diffusion testing and chest X-ray had been performed and gave no evidence of cardiac or pulmonary dysfunction. Laboratory results showed elevated creatine kinase (CK), transaminases and positive Severe acute respiratory syndrome coronavirus type 2 (SARS-COV-2) immunoglobulins of class M and G (IgM and IgG). A cardiac magnetic resonance imaging (MRI) showed no signs indicative of post-COVID-19 myocarditis, displaying a regular sized left ventricle with mild septal hypertrophy and normal cardiac output. The patient reported to not have noticed any kind of muscular weakness, myalgia, stiffness or muscular atrophy prior to COVID-19 infection. However, elevated CK (up to 2,500 U/l) had been noted on routine blood screens before but had not triggered neurological or further work-up. The patient reported muscular weakness most pronounced in legs and feet upon low physical exertion persisting after his COVID-19 disease. There was no myalgia at rest, during or after exercise. Medical history included allergic rhinoconjunctivitis and restless legs syndrome (RLS) and there was no intake of regular medication or known substance abuse. The diagnosis of RLS has been made about 20 years ago. The patient reported an urge to move his legs especially in the evening and at night with occasional sleep disturbance and alleviation upon movement. Symptomatic therapy has not been necessary according to our patient. Other unintended movement disorders throughout daytime or sleep disorders have not been reported or observed by our patient. Regarding the precise description of the patient’s complaints, all obligatory criteria are fulfilled to make a diagnosis of RLS (referring to the International RLS Study Group). We did not interpret his RLS as prodromal chorea. There was no family history for neuromuscular/neurological diseases. The mother of the patient had difficulties walking. She had a long-known spinal stenosis, but clinical examination showed normal reflex status, no muscle wasting, no fasciculation and no pareses. The neurological examination of our patient revealed slight weakness in abduction of the right hip (4/5), in dorsal extension and plantar flexion of both feet (4/5) and atrophy of the left calf muscle with regular muscle strength in the upper limbs. Figure 1B shows difference in calf circumference with a slimmer left lower leg and atrophy of the gastrocnemius muscle. No permanent sensory deficits were reported and clinical examination revealed mild reduction of vibration sense in the right lower leg (3/8 right malleolus, 4/8 right knee) while deep tendon reflexes were intact. Cranial nerve status and speech were normal. A *flexible endoscopic evaluation of swallowing* assessment was normal with no signs of dysphagia. Gait testing revealed Trendelenburg sign on the right side and walking on tiptoes and heels was impeded. Balance was intact and no involuntary movement disorders were observed. There were no signs of formal or content-related cognitive abnormalities and a neuropsychological screening revealed unsuspicious results regarding common screening instruments (*Bayer Activities of Daily Living Scale* B-ADL (1,16), *Hospital Anxiety and Depression Scale* HADS-D (anxiety: 7, depression: 1), *Montreal Cognitive Assessment* MoCa (29/30), *Symbol Digit Modalities test* SDMT (58). Laboratory results showed elevation of CK at all different time points of consultation (max. 4,163 U/l; reference: <174 U/l), Glutamic oxaloacetic transaminase (GOT) (max. 138 U/l; reference: 10–50 U/l) and lactate dehydrogenase (LDH) (max. 590 U/l; reference: 135–225 U/l) which prompted further diagnostic work-up with regards to myopathy and muscular dystrophies. GOT and LDH can be co-elevated in muscular dystrophies but can also be elevated in primary cardiac and hepatic diseases and can hint towards early stages of cardiomyopathy or hepatopathy respectively. Interestingly, anti-PM-Scl100 antibodies were detected in a specific myositis panel. Other antinuclear antibodies, antineutrophil cytoplasmic antibodies and rheumatoid factors were negative. Blood group profile revealed blood group type A, Rhesus D positivity (CcD.Ee) and K-k+ (kk) type. Further immunohaematological characterization was not possible due to technical and logistical reasons at time of manuscript publication but is scheduled in due course. Whole body muscle MRI showed bilateral fatty and oedematous atrophy of adductors, hamstrings and both calves (Figure 1C). MRI of the lumbar spine showed multisegmental degeneration with spinal narrowing at L3/4 and L4/5 but no significant neural foraminal constrictions or myelopathy (Supplementary Figure S1A). Electrophysiological investigations revealed axonal polyneuropathy with motor and sensory involvement, fasciculations and pathological spontaneous activity but formally normal motor unit action potentials (MUPs) in left vastus muscle, left tibial anterior muscle and left gastrocnemius muscle. Evoked potentials indicated an afferent sensory disorder of the left arm and both legs. Biopsy of the right gastrocnemius muscle showed severe atrophy of skeletal muscle without signs of inflammation, rimmed vacuoles or ragged red fibres. No other specific structural or immunohistochemical abnormalities were detected (Figure 1D). Lumbar puncture was performed after first admission at our department (10 months after the patient’s COVID-19 disease). CSF standard analysis showed no abnormalities in total cell count or protein and no intrathecal immunoglobulin synthesis. CSF flow cytometry revealed a slight elevation of HLA-DR CD4^+^ (27.8%, reference <24%) and HLA-DR CD8^+^ cells (75.5%, reference <63,4%), a CD4/CD8 ratio of 1.4 and an elevation of natural killer cells (NKs) (5,8%) (Figure 2A). Multi-gene panel sequencing including common candidate genes for myopathy or muscular dystrophy revealed a novel hemizygous deletion (c.756del, p.Trp253Glyfs*15) of the *XK* gene (Figure 2B). In order to examine its impact, we performed FACS analysis of blood which revealed negativity for Kell (K) and a reduced density of Cellano (k) antigen on the patient’s erythrocytes in comparison to healthy controls (Figure 2C). Real-time PCR revealed no restriction of *KEL* gene expression in our patient (Figure 2D). For an overview of clinical work-up of our patient, please see Supplementary Figure 1B.

## Discussion

Here, we present a case of MLS with a novel deletion in the *XK* gene.

Dominant features at presentation were raised CK, muscular weakness and atrophy, decreasing physical performance and exercise intolerance which the patient had only noted after COVID-19 disease. However, thorough work-up revealed longer-standing changes including neuropathy, muscle atrophy with fatty involution and raised CK, indicating longer-standing neuropathy and myopathy most likely due to the underlying genetic defect in the *XK* gene. Routine myositis screening revealed positive anti-PM-Scl100 antibodies which can be associated with overlapping polymyositis, scleroderma (Reichlin et al., 1984; Mahler M et al., 2005) or other autoimmune diseases (Lackner et al., 2020) and had triggered muscle biopsy for exclusion of myositis prior to the genetic diagnostic work-up. In light of absence of other antibody-positivity and changes indicative for inflammatory muscle disease in MRI and muscle histopathology, we interpreted these antibody findings as false positive/non-specific.

The identified novel deletion in *XK* leads to a frameshift and exchange of tryptophan (Trp) to glycine (Gly) at position number 253, resulting in a premature termination of the amino acid chain. The identified change is highly conserved, rare and has not yet been reported in the literature. Segregation analysis revealed the same deletion in the patient’s mother, identifying her as heterozygous carrier.

In order to analyse potential pathogenicity of the novel deletion we performed FACS which revealed reduced expression of k antigen in our patient compared to healthy controls. Negativity for the Kell (K) antigen has been shown to not be related to *XK* variation versus wildtype (Allen et al., 1961) but is most common in the overall population (92.5%) (Ristovska et al., 2022). We then investigated whether the *KEL* gene expression was diminished on RNA level. Real-time PCR showed no restriction of *KEL* specific RNA, indicating that the downregulation of Kell antigen expression associated with the identified *XK* deletion must be impeded in a later step of protein or antigen presentation. Russo *et al.* identified the assembly and transfer mechanisms of Kell and XK protein in an *in vivo* system (Russo et al., 1998). Physiologically, Kell and XK protein occur as a dimer complex, linked by a disulphide bond between cysteine (Cys) molecules, Kell Cys^72^ and XK Cys^347^, which is formed in the endoplasmic reticulum (ER) (Russo et al., 1998; Russo et al., 1999). It is unknown whether pathogenic variants in *XK* impact on the formation or stability of this disulphide bond. After assembly in the ER, the complex is transferred to the cell’s membrane and the Golgi compartment. The transport and expression of recombinant Kell and XK protein in *CV-1 in origin carrying SV40* (COS) cells in *in vivo* experiments appeared to be independent of the other protein (Russo et al., 1999), but clinical reports have shown that Kell protein expression and Kell antigen presentation are significantly diminished in patients with defect or loss of the XK protein. Intracellular proteins or enzymes that are involved in Kell/XK complex formation, transport and presentation on erythrocytes’ membranes have not been identified yet. It hence remains of great interest to further analyse intracellular processes and identify differences in patients with physiologically intact XK protein and in patients with pathogenic *XK* gene changes.

Exacerbation or, as in our case, subjective onset of clinical symptoms after infection (as in this case SARS-CoV-2) has to our knowledge not been investigated before in patients with MLS. For our patient, the onset of initially unspecific symptoms such as fatigue, decrease of physical performance and perception of unspecific muscle weakness was subjectively referred to COVID-19 disease. Consequently, he was admitted under presumption of post-COVID-19 syndrome. He hence underwent thorough diagnostic work-up, including examination of CSF where routine parameters revealed no abnormalities. However, CSF flow cytometry revealed elevation of HLA-DR^+^CD4^+^ and HLA-DR^+^CD8^+^ cells and elevation of NKs. HLA-DR^+^CD4^+^ and HLA-DR^+^CD8^+^ cells represent activation of T-lymphocytes in the central nervous system (CNS). Activated lymphocytes and NKs in the CSF are associated with immunological processes and have been found in autoimmune and inflammatory CNS diseases (Strunk et al., 2018; Gross et al., 2021). Here, the finding of elevated activated T cells and NKs indicate an immune-cell-mediated response in the CNS which *might* trigger a greater susceptibility to perception of muscular weakness and reduced physical strength after COVID-19 disease that then led to subjective notification of first symptoms of *XK*-disease in our patient. However, further analyses including CSF flow cytometry in patients with pathogenic variants in *XK* with or without prior SARS-CoV-2 infection are warranted.

Our patient displayed clinically dominating muscular weakness and neuropathy with lack of acanthocytosis, hepatopathy, cardiomyopathy or cognitive and psychiatric impairment to date. We hence wondered whether the location of the reported deletion correlates with specific clinical symptoms and if pathogenic changes in close proximity to the herein identified novel deletion might show clinical resemblance. Therefore, we performed a systematic analysis of patients reported in the literature with pathogenic *XK* gene changes, including nucleotide changes, insertions, deletions and gross deletions of *XK* gene. We enquired PubMed searching for the following keywords: *XK*, McLeod syndrome, gene variants/changes, deletion, insertion, missense mutation; as well as cited manuscripts in the respective publications. We only included patients where sufficient clinical/phenotypic data was available for clinicogenetic correlation which left us with 44 cases amenable to our analysis. Supplementary Table S1 gives an overview of the observed *XK* variants and their clinical manifestation. The spectrum of respective clinical symptoms could not be assigned to a specific type or localization of reported variants only. However, patients with pathogenic variants in exon 3 and in close proximity to the novel deletion of our patient (c.756del, p.Trp253Glyfs*15) did manifest with neuropathy and myopathy, frequently displaying areflexia, elevated CK and acanthocytosis without hepatopathy or cardiomyopathy. Neuropsychiatric symptoms and movement disorders were variably present in a subset of these.

As previously reported in the literature, symptoms may manifest successively over time and it cannot be ruled out that cognitive decline, a movement disorder or acanthocytosis might appear over time in our patient, strengthening the importance of follow-up exams in rare neurogenetic conditions. Informing and explaining the diagnosis of *XK* disease to our patient, we called his attention to possible manifestation of further neurological symptoms in the future, the risk of transfusion reactions and multi-systemic manifestation such as cardiomyopathy. Following our patient up, we will focus on his motor and sensory functions, possible emergence of movement disorders and his cognitive and emotional state. Additionally, cardiac MRI showed mild septal hypertrophy in our patient and cardiomyopathy can be a prominent feature of MLS, thus cardiac follow-up examination and practice management will be of high importance as well as proactive information and avoidance of potential elevated risk of transfusion reactions in this disease.

## Conclusion

Here, we report a novel deletion in *XK* in a patient with elevated creatine kinase, signs of myopathy and neuropathy as well as raised levels of activated T-lymphocytes in CSF. We performed a clinicogenetic review of a selection of reported genetically confirmed MLS cases in the literature and confirmed the broad multisystemic, but individually variable phenotypic presentation that cannot be predicted by location or type of genetic variant alone. We showed that the *XK* deletion in our patient results in diminished expression of the Kell blood group antigen Cellano (k), however, real-time PCR revealed no restriction of *KEL* gene expression on RNA level. It remains of great interest to identify the exact pathogenic mechanisms of Kell blood group antigen expression in the presence of XK protein and the impact of pathogenic *XK* gene variation leading to clinical manifestation in future studies of this rare disease.

## Data Availability

The datasets presented in this study can be found in online repositories. The names of the repository/repositories and accession number(s) can be found in the article/Supplementary Material.

## Supplementary material

SUPPLEMENTARY FIGURE S1 Diagnostic work-up of our patient. **(A)** MRI of lumbar spine. Multisegmental degeneration with spinal narrowing at L3/4 and L4/5 (white arrow) without significant neural foraminal constrictions or myelopathy. Abbreviations: CK, creatine kinase; MRI, magnetic resonance imaging. **(B)** Timeline of the diagnostic work-up of our patient.

## Abbreviations

ACMG, American College of Medical Genetics and Genomics; cDNA, Complementary desoxyribonucleic acid; CK, Creatine kinase; CNS, Central nervous system; COS cells, *CV-1 in origin carrying SV40* cells; COVID-19, Coronavirus disease 2019; CSF, Cerebrospinal fluid; Cys, Cysteine; ER. Endoplasmic reticulum; FACS, Fluorescence-activated Cell Sorting; Gly, Glycine; GOT, Glutamic oxaloacetic transaminase; Ig, Immunoglobulin; K, Kell antigen; k, Cellano antigen; LDH, Lactate dehydrogenase; MRI, Magnetic resonance imaging; MUP, Motor unit action potential; NK, Natural killer cell; PCR, Polymerase chain reaction; RBC, Red blood cell; Rh, Rhesus; RNA, Ribonucleic acid; SARS-CoV-2, Severe acute respiratory syndrome coronavirus type 2; STIR, Short Tau Inversion Recovery sequence; Trp, Tyrptophane; U/l, Unit per liter
