# Supplementary figures and images for: Case report: Clinical, genetic and immunological characterization of a novel XK variant in a patient with McLeod syndrome
Source: Front Genet. 2024 Aug 21;15:1421952. doi: 10.3389/fgene.2024.1421952 (PMC11371627; doi:10.3389/fgene.2024.1421952)

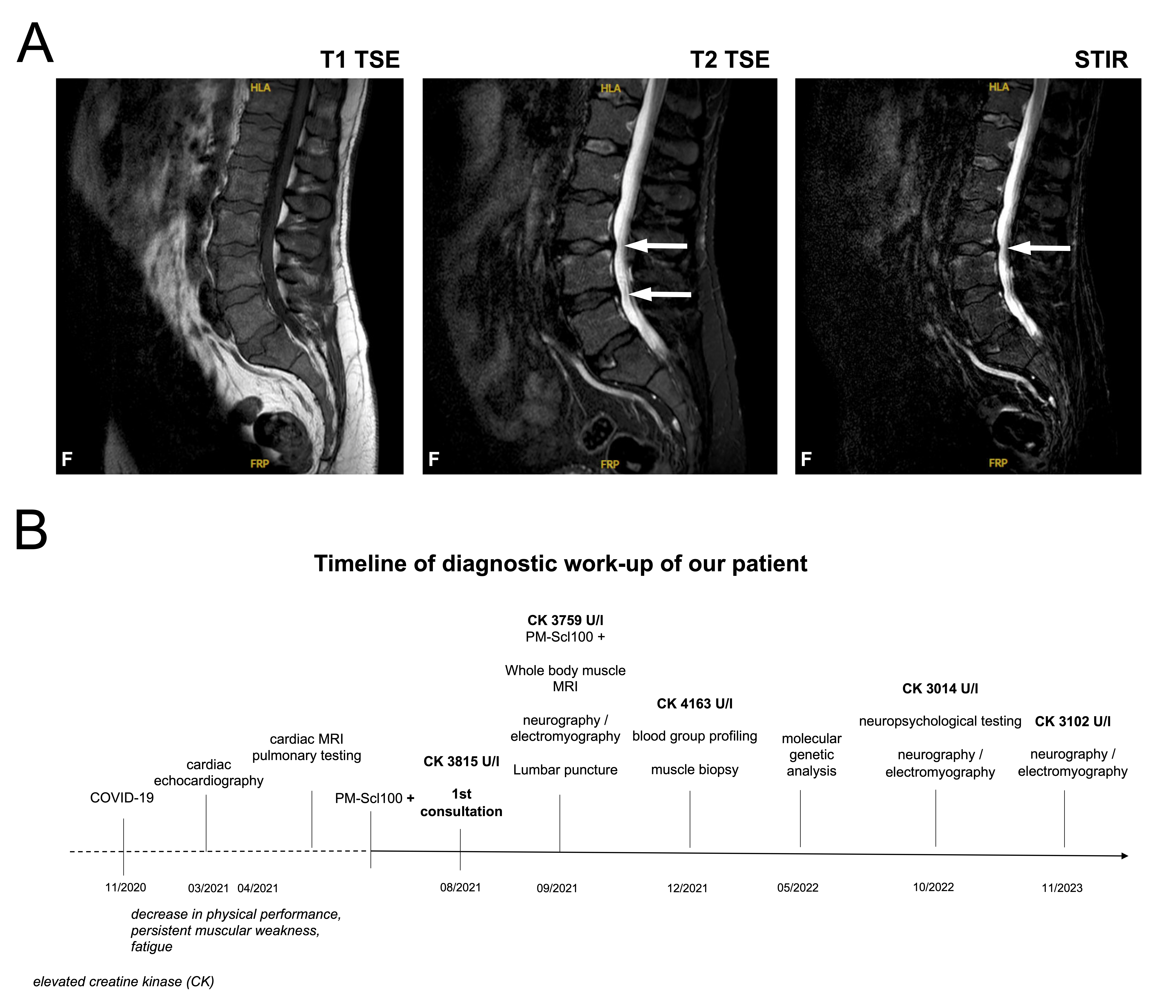

Supplement: Supplementary file 1 [file Image1.JPEG]
