# Supplementary material for: Case report: Clinical, genetic and immunological characterization of a novel XK variant in a patient with McLeod syndrome
Source: Front Genet. 2024 Aug 21;15:1421952. doi: 10.3389/fgene.2024.1421952 (PMC11371627; doi:10.3389/fgene.2024.1421952)
Supplement: Supplementary file 3 [file DataSheet1.DOCX]

**Supplementary Data 1**

**Methods**

Molecular genetic analysis: Genetic analysis of blood was performed using a customised myopathy multi-gene panel (Twist Version 1 analysed with varvis® (version 1.19)) covering candidate genes for myopathy and muscular dystrophy, including the following genes.: *ACADVL ACTA1 ACVR1 AMPD1 ANO5 ATP2A1 BAG3 BIN1 CAPN3 CASQ1 CAV3 CCDC78 CFL2 CHCHD10 CLCN1 CNTN1 COL12A1 COL6A1 COL6A2 COL6A3 CPT2 CRPPA CRYAB DAG1 DES DMD DNA2 DNAJB6 DNM2 DPM3 DYSF EMD FHL1 FKBP14 FKRP FKTN FLNC GAA GMPPB GNE HACD1 HINT1 HNRNPA1 HNRNPA2B1 HNRNPDL ISCU KBTBD13 KLHL40 KLHL41 KLHL9 LAMA2 LAMP2 LDB3 LMNA LMOD3 LPIN1 MATR3 MEGF10 MSTN MTM1 MTMR14 MYF6 MYH14 MYH2 MYH7 MYOT NEB OPA1 ORAI1 PABPN1 PGK1 PHKA1 PLEC PNPLA2 POLG POLG2 POMGNT1 POMK POMT1 POMT2 PUS1 PYGM RRM2B RYR1 SCN4A SELENON SGCA SGCB SGCD SGCG SIL1 SPEG STAC3 STIM1 SUCLA2 TCAP TIA1 TK2 TNNT1 TNPO3 TPM2 TPM3 TRAPPC11 TRIM32 TTN TWNK VCP VMA21 XK YARS2*

Gene expression analysis: Analysis of *KEL* gene expression was performed by real-time polymerase chain reaction (real-time PCR) using a validated expression assay (TaqMan™ Gene Expression Assay) with specific primers and StepOne™ software (version 2.2.2. Applied Biosystems 2011) for RNA quantification analysis.

**Real-time PCR (RT-PCR)**

Real-time PCR, also known as quantitative PCR (qPCR) is used to amplify and quantitate specific DNA / RNA sequences in real time. After an initial phase of PCR performance, amplification conditions reach an optimum state with an exponential increase of amplified DNA / RNA (for up to 30 amplification cycles). At the beginning of each amplification cycle, DNA/ RNA concentration can be measured in so-called cycle threshold (Ct value) which gives information on the amount of amplified DNA / RNA in a sample. qPCR allows quantification of DNA / RNA in real time after every amplification cycle.

The ΔΔCt method is used for direct comparison of gene expression and DNA / RNA quantity following qPCR amplification of two or more samples. The ΔΔCt method is performed with a focus on relative quantification and comparison of qPCR data. After calculating the difference of cycle thresholds (Ct values) of two different samples / groups, it gives information on the relative expression of DNA/ RNA as n-fold expression of a target gene, also represented as 2 −ΔΔCt.

**Primers used for cDNA sequencing of the *KEL* gene.**

| ***Name*** | ***Direction*** | ***Sequence (5’ – 3’)*** |
| --- | --- | --- |
| 1. ***Amplification*** |  |  |
| KEL 1s2 | forward | GCGAAAGAGCAGCAGAAGTG |
| KEL 1as1 | reverse | GAAGCCAAGTGCCAGCTTTT |
| 1. ***Sequencing*** |  |  |
| K-Seq_cDNA_211s | forward | ATGGAAGGTGGGGACCAAAGTG |
| K-Seq_cDNA_441s | forward | TGAGACATCTGTGTGTTTGG |
| K-Seq_cDNA_750s | forward | CATCTCTGGTAAATGGACTTCC |
| K-Seq_cDNA_1025s | forward | CTTCCTTGTCAATCTCCATCAC |
| K-Seq_cDNA_1390s | forward | GAACTGACAGAGCAACCAC |
| K-Seq_cDNA_1831s | forward | GCTTACTATTCGGTATCTGACC |
| K-Seq_cDNA_2249s | forward | TGATGTGTAGGAAGCCCAG |
| K-Seq_cDNA_400as  K-Seq_cDNA_698as  K-Seq_cDNA_2096as | reverse  reverse  reverse | GCACAGAAAAACAAAGGAGC  GCTTCAATGGCAAGTGTATCC  GAGTCATTGAAGGAGGTTCTG |
|  |  |  |

**Preparation of the PCR reaction mix**

1. cDNA samples were kept on ice and inverted and gently vortexed before mixture.
2. Preparation of the Master mix included

5 µl Master Mix (2x)

0.5 µl TaqManTM Gene Expression Assay (20X)

3.5 µl Nuclease-free water

1. 9 µl of the Master mix were distributed per well on to a well-plate. 1µl of cDNA sample was added to each well. 1µl of nuclease-free water was used as negative control.
2. The plate was sealed with a MicroAmp™ Optical Adhesive Film and vortexed briefly.
3. The plate was loaded on to the real-time PCR instrument and run in the StepOne™ system with fast cycling mode in the following steps

Step Temperature Time Cycles
UNG incubation 50°C 2 min 1

Enzyme activation 95°C 20 sec 1

Denaturation 95°C 1 sec 40

Anneal / Extension 60°C 20 sec 40
